# Associations Between Patient Satisfaction with Pharmacist Services, Social Support, and Medication Adherence in Chronic Dermatological Conditions: A Cross-Sectional Study

**DOI:** 10.3390/healthcare14152346

**Published:** 2026-08-01

**Authors:** Nicoleta Cirstea, Delia Mirela Tit, Anamaria Lavinia Purza, Mirela Marioara Toma, Ada Radu, Gabriela S. Bungau, Ruxandra-Cristina Marin, Călin Muntean, Radu Dumitru Moleriu

**Affiliations:** 1 Doctoral School of Biomedical Sciences, Faculty of Medicine and Pharmacy, University of Oradea, 410087 Oradea, Romania; carstea.nicoleta@yahoo.com (N.C.); adaradu@uoradea.ro (A.R.); gbungau@uoradea.ro (G.S.B.); marin.ruxandracristina@student.uoradea.ro (R.-C.M.); 2 Department of Pharmacy, Faculty of Medicine and Pharmacy, University of Oradea, 410028 Oradea, Romania; mtoma@uoradea.ro; 3 Department of Pharmacology, Clinical Pharmacology and Pharmacotherapy, Faculty of Medicine, “Carol Davila” University of Medicine and Pharmacy, 050474 Bucharest, Romania; 4 Department III, Functional Science, Discipline of Medical Informatics and Biostatistics, “Victor Babes” University of Medicine and Pharmacy, 300041 Timisoara, Romania; cmuntean@umft.ro (C.M.); radu.moleriu@umft.ro (R.D.M.)

**Keywords:** medication adherence, patient satisfaction, community pharmacy, dermatology, quality of life, patient-reported outcomes, cross-sectional study, Romania

## Abstract

**Background/Objectives**: Chronic dermatological conditions often require long-term treatment and can substantially impair quality of life. This study examined associations among satisfaction with pharmacist services, medication adherence, social support, and dermatology-related quality of life in adults with chronic skin disease. **Methods**: A cross-sectional analysis of baseline data was conducted in 220 adults recruited from community pharmacies in Romania. Medication adherence was assessed using the dermatology-adapted Medication Adherence Report Scale-5 (MARS-5), dermatology-related quality of life using the Dermatology Life Quality Index (DLQI), and satisfaction with pharmacist services using the Romanian Patient Satisfaction with Pharmacist Services Questionnaire 2.0 (PSPSQ-2). Analyses included correlations, multivariable regression, mediation, and subgroup analyses. **Results**: Participants were predominantly female (66.4%) and urban residents (65.5%); eczema, acne, and psoriasis were the most frequent diagnoses. Baseline scores indicated high adherence (MARS-5: 21.50 ± 4.19) and satisfaction (PSPSQ-2: 62.92 ± 9.57), alongside substantial quality-of-life impairment (DLQI: 11.12 ± 7.86). Higher satisfaction with pharmacist services was associated with better medication adherence in the primary adjusted model (B = 0.076, 95% CI: 0.017–0.135; *p* = 0.011). After inclusion of perceived social support, this association was attenuated (*p* = 0.053). Perceived social support was independently associated with adherence, and the explained variance increased from 13.3% to 28.0%. **Conclusions**: Higher satisfaction with pharmacist services was associated with better adherence, although this association was attenuated after perceived social support was included. These findings support considering the psychosocial context when designing and evaluating community-pharmacy adherence support.

## 1. Introduction

A defining characteristic of many chronic dermatological diseases is their chronic and relapsing course, frequently requiring prolonged treatment with topical agents, systemic therapies, phototherapy, or combinations thereof. Despite the availability of effective treatments, medication adherence remains a major challenge across common skin conditions, including psoriasis, acne, atopic dermatitis, chronic urticaria, and fungal skin diseases. Non-adherence is influenced by multiple patient-, disease-, treatment-, and healthcare-related factors, including treatment complexity, concerns regarding adverse effects, inconvenience of topical regimens, insufficient patient education, unrealistic treatment expectations, and inadequate patient–provider communication. Consequently, adherence is increasingly recognized as a multifactorial behavioural phenomenon requiring multidisciplinary strategies to support patients throughout long-term treatment. Poor adherence contributes to treatment failure, disease recurrence, impaired quality of life, and increased healthcare utilization [1]. Health literacy and patients’ beliefs regarding treatment necessity, concerns about medication safety, and confidence in treatment effectiveness further influence adherence behaviour, highlighting the importance of patient-centred approaches that address both informational and motivational barriers to treatment adherence [2,3].

Medication adherence in dermatology is particularly challenging because treatment often involves prolonged topical therapy, complex application regimens, delayed clinical improvement, and concerns regarding topical corticosteroids and other treatment-related adverse effects. These disease-specific barriers may reduce treatment persistence and emphasize the need for ongoing patient education and counselling, highlighting an important opportunity for community pharmacists to support adherence [1,4].

Community pharmacists are well positioned to address several of these barriers because they remain accessible throughout long-term treatment and frequently interact with patients when dispensing prescriptions, supplying medication refills, or providing treatment-related advice. Pharmaceutical care has been defined by the Pharmaceutical Care Network Europe as the pharmacist’s contribution to the care of individuals in order to optimize medicines use and improve health outcomes. In operational terms, this patient-centred approach involves identifying patients’ medication-related needs, detecting and resolving drug-related problems, providing education and counselling, and monitoring the outcomes of care [5]. In chronic dermatological conditions, these principles translate into reinforcing treatment instructions, supporting the correct application of topical therapies, identifying practical and perceptual adherence barriers, monitoring medication-related problems, and referring patients for medical reassessment when appropriate [4,6].

International evidence from different healthcare settings supports the expansion of community pharmacists’ roles in chronic disease management. A mixed-methods systematic review conducted across low- and middle-income countries identified health education, medication review, referral, screening, point-of-care testing, and support for chronic disease self-management among the most frequently reported community-pharmacy services [7]. A randomized controlled trial conducted in Saudi Arabia showed significantly higher medication adherence and patient satisfaction with a community pharmacy-based medication therapy management programme than with standard care, together with a reduction in identified drug-related problems over follow-up; however, the between-group difference in HbA1c was not statistically significant [8]. A Saudi observational study evaluated satisfaction with pharmaceutical care services and medication adherence among patients with chronic diseases [9], whereas a Lebanese cross-sectional study examined associations of pharmacy services and pharmacist–patient relationships with health-related quality of life [10]. Dermatology-specific evidence remains more limited and has focused primarily on medication counselling, treatment monitoring, adherence barriers, access to medications, and the correct use of topical treatments [4].

Existing consensus recommendations and professional practice resources describe complementary roles for pharmacists in dermatological care. At the disease-specific level, a Delphi consensus between dermatologists and hospital pharmacists regarding atopic dermatitis emphasized coordinated follow-up, assessment of treatment response, and effective communication between dermatology and hospital-pharmacy services [11].

Professional resources developed by the British Association of Dermatologists recognize the contribution of pharmacists working in both community and secondary-care dermatology settings and provide role descriptions and training resources for specialist practice [12]. At the community-pharmacy level, a recent cross-sectional study examining counselling on treatment administration, duration, storage, adverse effects, and monitoring requirements identified educational needs and variability in pharmacists’ knowledge and practices concerning topical medications [6].

Within contemporary healthcare systems, patient-reported measures are increasingly recognized as key indicators of healthcare quality and patient-centred care. Patient-reported outcome measures (PROMs) assess patients’ perceptions of health status, symptoms, functioning, and quality of life, whereas patient-reported experience measures (PREMs) evaluate experiences with healthcare services and interactions with healthcare professionals. Together, these measures provide complementary perspectives that extend beyond traditional clinical indicators and support healthcare evaluation, quality improvement, and identification of unmet patient needs [13].

Within dermatology, patient-reported outcomes are particularly important because objective clinical severity does not always correspond closely with patients’ perceived disease burden. Chronic skin diseases may adversely affect self-esteem, social participation, emotional well-being, occupational functioning, interpersonal relationships, and overall quality of life [14]. Consequently, dermatology-specific quality-of-life measures have become indispensable tools for evaluating treatment effectiveness and understanding the broader impact of skin diseases from the patient’s perspective [14,15,16].

In community-pharmacy practice, satisfaction with pharmacist services reflects patients’ perceptions of the care received and their interactions with pharmacists. The Patient Satisfaction with Pharmacist Services Questionnaire 2.0 (PSPSQ-2) captures this experience across three domains: perceived quality of care, the patient–pharmacist relationship, and overall satisfaction [17,18]. Medication adherence represents treatment-related behavior, whereas dermatology-specific quality of life represents a patient-reported health outcome. Perceived social support has been associated with medication adherence in patients with chronic disease [19] and with self-management through psychological resilience and health empowerment in older patients with chronic conditions [20]. Social determinants, including interpersonal and social-support factors, have also been linked to health-related quality of life across chronic disease populations [21]. Evaluating these constructs together may therefore provide a more comprehensive account of pharmaceutical care than examining satisfaction, adherence, or quality of life separately. However, because their relationships may be bidirectional and influenced by unmeasured clinical and psychosocial factors, associations identified in cross-sectional data should not be interpreted causally.

To our knowledge, evidence examining patient satisfaction, medication adherence, perceived social support, and dermatology-specific quality of life concurrently in patients with chronic dermatological conditions remains limited, particularly in routine community-pharmacy settings. Within Central and Eastern Europe, clinical pharmacy services remain underdeveloped or underutilized in several countries [22], while Romanian research has documented a substantial disease-related quality-of-life burden among patients with psoriasis [23]. The Romanian version of the PSPSQ-2 was recently translated, culturally adapted, and psychometrically validated in adults with chronic dermatological conditions, providing an appropriate instrument for assessing satisfaction with pharmacist services in this population [17]. The present study therefore provides an integrated evaluation of these dimensions in Romanian adults with chronic dermatological conditions recruited through community pharmacies. Its primary objective was to examine the associations among satisfaction with pharmacist services, medication adherence, perceived social support, and dermatology-specific quality of life. Secondary objectives were to identify factors independently associated with medication adherence and dermatology-specific quality of life and to explore whether medication adherence statistically mediated the association between satisfaction with pharmacist services and dermatology-specific quality of life.

## 2. Materials and Methods

### 2.1. Study Design, Setting and Participants

This study presents a cross-sectional analysis of baseline data collected as part of a broader research project evaluating pharmacist-related patient-reported outcomes in chronic dermatological conditions. Data were collected between January 2025 and January 2026 in five community pharmacies belonging to national pharmacy chains located in Oradea, Romania. The participating pharmacies constituted a convenience sample and were selected based on their proximity to hospitals and dermatology outpatient clinics, which facilitated access to patients receiving ongoing dermatological care, and their willingness to participate in the study. Only baseline data were included in the present analysis.

Within each participating pharmacy, eligible patients were recruited consecutively during routine pharmacy visits. All individuals meeting the eligibility criteria during the recruitment period were invited to participate. Potentially eligible individuals were identified when presenting with a dermatologist-issued prescription or when requesting advice, treatment, medication refills, or counselling related to a chronic dermatological condition. Eligibility was based on a patient-reported physician diagnosis and current receipt of dermatological treatment. When a dermatologist-issued prescription was available at recruitment, the pharmacist used it to corroborate the reported diagnosis through the prescribed treatment. Pharmacists received standardized instructions regarding eligibility criteria, participant information, and questionnaire administration.

#### 2.1.1. Inclusion Criteria

Eligible participants were adults aged 18 years or older who reported having a physician-diagnosed chronic dermatological condition, were currently receiving dermatological treatment and had received pharmacist consultation. Chronic dermatological conditions were defined as physician-diagnosed skin diseases requiring ongoing management through pharmacological treatment and/or regular medical follow-up. Participants were also required to understand Romanian and to provide written informed consent.

#### 2.1.2. Exclusion Criteria

Individuals were excluded at the participant level if they were unable to complete the study questionnaires independently. At the analytical stage, returned questionnaires were excluded when incomplete data precluded calculation of the study outcome scores.

#### 2.1.3. Final Analytical Sample and Sample-Size Considerations

A total of 300 questionnaires were distributed, of which 233 were returned (response rate: 77.7%). After exclusion of incomplete responses, 220 questionnaires were included in the final analytical sample.

The sample size was fixed by the number of participants with complete baseline data available from the broader research project; therefore, no separate a priori sample-size calculation was conducted specifically for the present secondary analysis. A sensitivity analysis for the omnibus F-test of the largest multivariable linear regression model was performed using base R functions within the R console embedded in JASP version 0.19.3. With N = 220, seven tested predictors, α = 0.05, and 80% statistical power, the minimum detectable effect size was Cohen’s f^2^ = 0.067.

### 2.2. Outcome Measures and Instruments

Three patient-reported instruments were administered at baseline: the Medication Adherence Report Scale-5 (MARS-5), the Dermatology Life Quality Index (DLQI), and the Patient Satisfaction with Pharmacist Services Questionnaire 2.0 (PSPSQ-2).

Medication adherence was assessed using a dermatology-adapted version of the 5-item Medication Adherence Report Scale (MARS-5), a self-report measure designed to elicit patients’ reports of non-adherent medication-taking behaviors [24]. The scale includes five items assessing behaviors such as forgetting, altering, stopping, skipping or using less medication than prescribed. Each item was scored from 1 to 5, yielding a total score from 5 to 25, with higher scores indicating better self-reported adherence. For the present study, item wording was contextually adapted to refer to prescribed dermatological treatment, including topical and/or systemic therapies. Because this dermatology-adapted wording has not undergone full psychometric validation, internal consistency was assessed in the present sample.

Dermatology-related quality of life was assessed using the Dermatology Life Quality Index (DLQI), a widely used dermatology-specific patient-reported outcome measure [25]. The instrument comprises 10 items covering symptoms and feelings, daily activities, leisure, work or school, personal relationships, and treatment-related burden. Total scores range from 0 to 30, with higher scores indicating greater quality-of-life impairment. Responses marked as “not relevant” were scored as 0 according to the scoring instructions. Established DLQI banding was used for interpretation: 0–1, no effect; 2–5, small effect; 6–10, moderate effect; 11–20, very large effect; and 21–30, extremely large effect on the patient’s life [26].

The Romanian version was used under licence from Cardiff University (Licence ID: CUQoL2470).

Satisfaction with pharmacist services was assessed using the Romanian version of the Patient Satisfaction with Pharmacist Services Questionnaire 2.0 (PSPSQ-2), originally developed by Sakharkar et al. [18]. The instrument contains 19 items rated on a 4-point Likert scale, generating a total score ranging from 19 to 76, with higher scores indicating greater satisfaction. The psychometric properties of the Romanian PSPSQ-2 in this cohort have been reported previously [17]. Because validation analyses demonstrated excellent reliability and supported a dominant general satisfaction factor, the total PSPSQ-2 score was used in the present study.

Perceived social support was assessed using a single self-reported item included in the baseline questionnaire. Participants rated their overall level of social support (e.g., from family, friends, or the community) on a five-category scale ranging from none to very good, with higher values indicating greater perceived social support.

In addition to the study instruments, sociodemographic, clinical, and healthcare-access characteristics were collected using a structured baseline questionnaire. These included age, gender, residence, education level, occupation, marital status, dermatological diagnosis, disease duration, treatment type, frequency of healthcare visits, access to care, and health insurance status. Education was recorded in four ordered categories, whereas treatment type was recorded as topical therapy only, systemic therapy with or without topical treatment, or other treatment/phototherapy.

### 2.3. Statistical Analysis

Statistical analyses were performed using JASP version 0.19.3. A two-tailed *p*-value < 0.05 was considered statistically significant. Continuous variables were summarized as mean ± standard deviation (SD) and median (interquartile range, IQR), while categorical variables were presented as frequencies and percentages.

Questionnaire scores were calculated according to the respective scoring manuals. For the DLQI, “not relevant” responses were scored as 0 in accordance with standard scoring procedures. Ceiling effects were calculated as the proportion of participants achieving the maxi-mum possible score. Internal consistency was assessed using Cronbach’s alpha for the dermatology-adapted MARS-5 and DLQI.

For descriptive presentation, perceived social-support responses were grouped as very good/good, moderate, and poor/none. For inferential analyses, the original five-level variable was entered as a single ordinal score, with higher values indicating greater perceived support. Education was retained as a four-level ordinal variable in the Spearman correlation analyses and was dichotomized for the multivariable regression and mediation analyses as university/postgraduate versus below-university education. Treatment type was dichotomized for the regression and mediation analyses as systemic treatment, either alone or combined with topical therapy, versus non-systemic treatment, comprising topical therapy only and other treatment/phototherapy. Below-university education and non-systemic treatment served as the reference categories.

Because the primary patient-reported outcome measures showed non-normal distributions, Spearman rank correlations were used to examine associations between satisfaction with pharmacist services (PSPSQ-2), medication adherence (MARS-5), dermatology-related quality of life (DLQI), perceived social support, and selected socio-demographic variables.

Multivariable linear regression analyses were performed to identify factors associated with medication adherence and dermatology-related quality-of-life impairment. Variables showing clinical relevance or significant bivariate associations were included in the models. Multicollinearity was assessed using variance inflation factors.

An exploratory cross-sectional mediation analysis was conducted to evaluate whether medication adherence mediated the association between satisfaction with pharmacist services and dermatology-related quality of life. Indirect effects were estimated using 20,000 bootstrap resamples and reported with bias-corrected percentile 95% confidence intervals. Standardized coefficients were reported, and *p*-values were derived from asymptotic maximum-likelihood z-tests. The mediation model was adjusted for age, residence, education, and treatment type.

Exploratory subgroup analyses according to residence, diagnosis, and education level were performed using Mann–Whitney U and Kruskal–Wallis tests, with Dunn’s post hoc comparisons where appropriate. Effect sizes were reported as rank-biserial correlations for Mann–Whitney tests and epsilon-squared values for Kruskal–Wallis analyses.

## 3. Results

### 3.1. Participant Characteristics

Participants were predominantly female (66.4%) and urban residents (65.5%). Nearly half had university-level education (47.3%), while 6.4% had postgraduate education. The most frequent diagnoses were eczema (31.4%), acne (24.5%) and psoriasis (23.2%), together accounting for 79.1% of the sample. Disease duration exceeded one year in 68.1% of participants.

Most participants were treated with topical therapy only (70.0%), whereas 26.8% received systemic therapy with or without topical treatment. Most respondents reported easy or very easy access to healthcare services (89.5%), had health insurance coverage (89.5%) and described their social support as good or very good (81.8%). Detailed baseline characteristics are shown in Table 1.

### 3.2. Descriptive Statistics of Primary Outcomes

Descriptive statistics for medication adherence, dermatology-related quality of life and satisfaction with pharmacist services are presented in Table 2. The mean MARS-5 score was 21.50 ± 4.19, with a median of 23 (IQR: 19–25), indicating high self-reported medication adherence. A substantial ceiling effect was observed, with 35.9% of participants achieving the maximum possible MARS-5 score.

The mean DLQI score was 11.12 ± 7.86, with a median of 11 (IQR: 4–17). Both the mean and median scores fell within the very large effect category according to established DLQI banding, indicating substantial dermatology-related quality-of-life impairment. As shown in Table 3, 39.5% of participants reported a very large effect and 12.3% an extremely large effect of their skin condition on daily life, while 13.2% reported no effect.

The mean PSPSQ-2 total score was 62.92 ± 9.57, with a median of 60 (IQR: 57–74), indicating high satisfaction with pharmacist services. A ceiling effect was also observed for PSPSQ-2, with 24.5% of participants achieving the maximum score. Because the Romanian PSPSQ-2 demonstrated a dominant general satisfaction factor, the total score was used as the satisfaction measure in subsequent analyses.

Internal consistency was good for the dermatology-adapted MARS-5 scale (Cronbach’s alpha = 0.891) and excellent for the DLQI (Cronbach’s alpha = 0.919). Corrected item-rest correlations ranged from 0.647 to 0.833 for MARS-5 and from 0.540 to 0.817 for DLQI. The PSPSQ-2 total score showed excellent internal consistency in the companion validation study (Cronbach’s alpha = 0.978), supporting its use as a global satisfaction measure.

### 3.3. Bivariate Associations Between Adherence, Satisfaction, and Quality of Life

Satisfaction with pharmacist services was positively correlated with medication adherence (*ρ* = 0.232, *p* < 0.001), indicating that higher PSPSQ-2 scores were associated with higher self-reported MARS-5 scores. Satisfaction was also positively correlated with DLQI scores (*ρ* = 0.319, *p* < 0.001). Because higher DLQI scores indicate greater quality-of-life impairment, this finding suggests that participants reporting greater dermatology-related burden also tended to report higher satisfaction with pharmacist services.

Medication adherence was positively correlated with DLQI scores (*ρ* = 0.234, *p* < 0.001), indicating higher adherence among participants reporting greater dermatology-related quality-of-life impairment. Among contextual variables, perceived social support showed the strongest association with adherence (ρ = 0.453, *p* < 0.001), substantially exceeding the correlations observed for residence (ρ = 0.288, *p* < 0.001) and education level (ρ = 0.137, *p* = 0.043). Urban residence was associated with higher adherence scores, while higher educational attainment was associated with modestly higher adherence.

**Figure 1:**
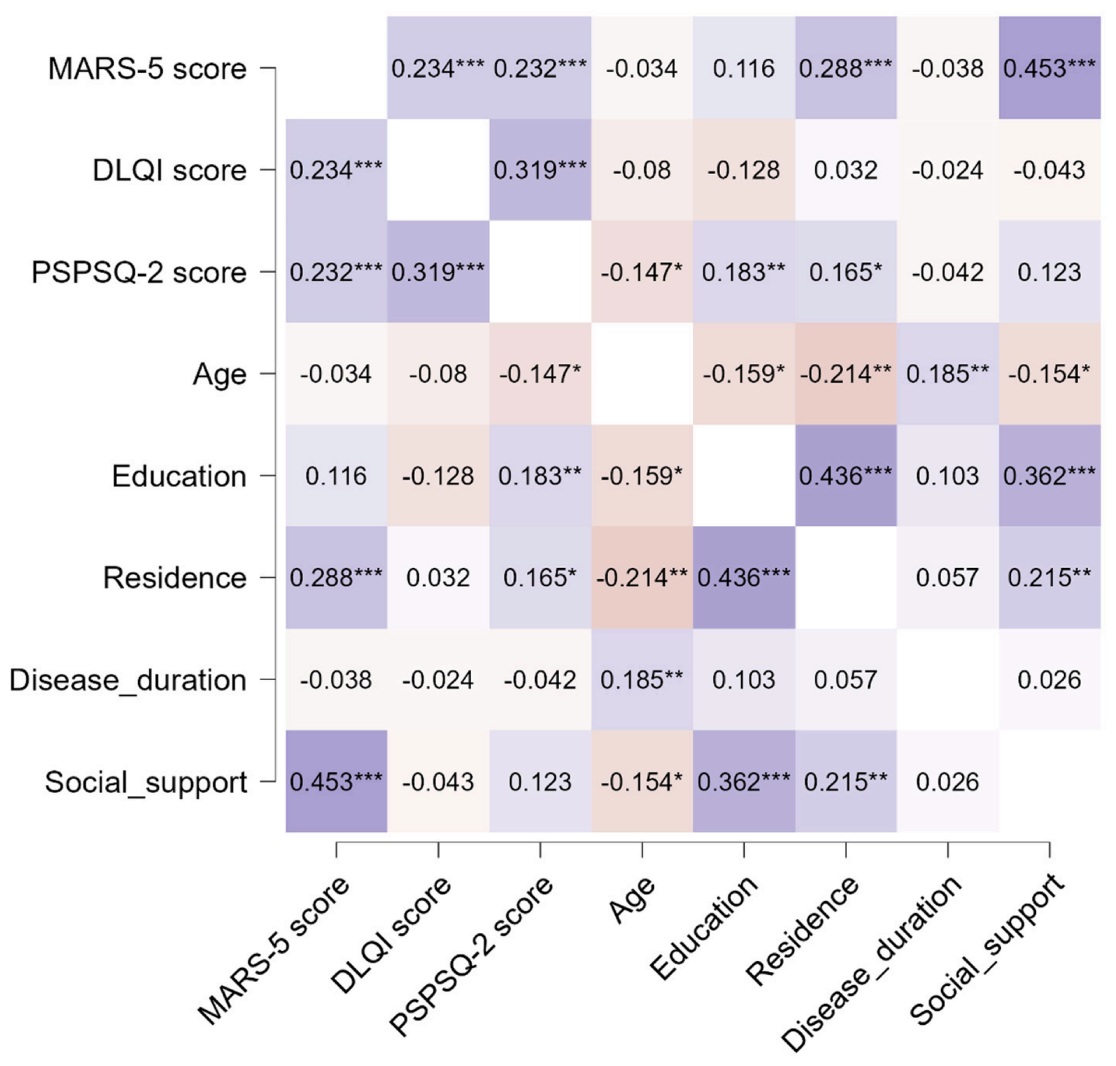
Spearman correlation heatmap for primary outcomes and selected socio-demographic/contextual variables. Positive correlations with DLQI indicate greater dermatology-related quality-of-life impairment. * indicates *p* < 0.05; ** indicates *p* < 0.01; *** indicates *p* < 0.001.

Satisfaction with pharmacist services was also associated with education level (ρ = 0.183, *p* = 0.006) and urban residence (ρ = 0.165, *p* = 0.014), whereas age showed a weak negative association with satisfaction (ρ = −0.147, *p* = 0.030). Significant positive correlations were observed between all three primary patient-reported measures; however, the positive direction of associations involving DLQI reflects greater quality-of-life impairment and should not be interpreted as better quality of life. Overall, the statistically significant correlations ranged from weak to moderate in magnitude. Correlations below |ρ| = 0.20 included education with adherence and satisfaction, residence with satisfaction, and age with satisfaction. Perceived social support showed the strongest association with medication adherence (ρ = 0.453). Table 4 presents statistically significant correlations involving the primary outcome measures, while the complete correlation matrix is shown in Figure 1.

### 3.4. Multivariable Regression Models for Medication Adherence

Two multivariable linear regression models were fitted with MARS-5 as the dependent variable. Model 1 examined the association between satisfaction with pharmacist services and medication adherence after adjustment for socio-demographic and clinical variables. Model 2 additionally included perceived social support, because this variable showed the strongest bivariate association with adherence.

In Model 1, the overall model was statistically significant (*F* = 5.467, *p* < 0.001) and explained 13.3% of the variance in MARS-5 scores (*R*^2^ = 0.133; adjusted *R*^2^ = 0.109). Urban residence was associated with higher adherence scores compared with rural residence (*B* = 2.182, 95% *CI*: 0.911 to 3.454, *β* = 0.248, *p* < 0.001). Higher satisfaction with pharmacist services was also associated with higher adherence scores (*B* = 0.076, 95% CI: 0.017 to 0.135, *β* = 0.174, *p* = 0.011), as was higher DLQI impairment (*B* = 0.081, 95% CI: 0.011 to 0.152, *β* = 0.153, *p* = 0.023). Age, education and gender were not significantly associated with MARS-5.

In Model 2, the addition of perceived social support increased the explained variance to 28.0% (*R*^2^ = 0.280; adjusted *R*^2^ = 0.256; *F* = 11.785, *p* < 0.001). Social support was the strongest independent correlate of adherence (*B* = 1.787, 95% *CI*: 1.251 to 2.323, *β* = 0.411, *p* < 0.001). Urban residence remained significantly associated with higher MARS-5 scores (*B* = 2.057, 95% *CI*: 0.894 to 3.219, *β* = 0.234, *p* < 0.001), as did DLQI *(B* = 0.094, 95% *CI*: 0.030 to 0.159, *β* = 0.177, *p* = 0.004). After adjustment for social support and other covariates, higher educational attainment was associated with slightly lower adherence scores (*B* = −0.601, 95% *CI*: −1.176 to −0.027, *β* = −0.143, *p* = 0.040). The association between PSPSQ-2 and MARS-5 was attenuated and no longer reached conventional statistical significance after perceived social support was included (*B* = 0.054, 95% *CI*: −0.005 to 0.108, *β* = 0.122, *p* = 0.053) (Table 5).

Collinearity diagnostics did not indicate problematic multicollinearity in either model, with all variance inflation factors ≤ 1.420. Together, these models suggest that satisfaction with pharmacist services was associated with medication adherence after adjustment for demographic and clinical factors. However, this association was attenuated after inclusion of perceived social support, which emerged as the strongest independent correlate of adherence.

### 3.5. Multivariable Regression Model for Dermatology-Related Quality of Life

A multivariable linear regression model was fitted with DLQI as the dependent variable to examine factors associated with dermatology-related quality-of-life impairment. The overall model was statistically significant (*F* = 5.399, *p* < 0.001) and explained 11.2% of the variance in DLQI scores (*R*^2^ = 0.112; adjusted *R*^2^ = 0.091).

Higher satisfaction with pharmacist services was positively associated with DLQI scores (*B* = 0.175, 95% *CI*: 0.066 to 0.284, *β* = 0.213, *p* = 0.002), indicating higher satisfaction scores among participants reporting greater dermatology-related quality-of-life impairment. Systemic treatment, compared with non-systemic treatment, was associated with higher DLQI scores (*B* = 3.110, 95% *CI*: 0.823 to 5.398, *β* = 0.176, *p* = 0.008). In contrast, higher education level was associated with lower DLQI scores (B = −1.508, 95% *CI*: −2.544 to −0.473, *β* = −0.192, *p* = 0.005). Age and gender were not significantly associated with DLQI (Table 6).

Collinearity diagnostics did not indicate problematic multicollinearity, with all variance inflation factors ≤ 1.089.

### 3.6. Exploratory Mediation Analysis

An exploratory mediation analysis was conducted to examine whether medication adherence mediated the association between satisfaction with pharmacist services and dermatology-related quality-of-life impairment. The model included PSPSQ-2 as the independent variable, MARS-5 as the mediator, and DLQI as the dependent variable, with adjustment for age, residence, education, and treatment type.

The total association between PSPSQ-2 and DLQI was statistically significant (β = 0.206, SE = 0.077, z = 2.676, *p* = 0.007, 95% bootstrap CI: 0.050 to 0.351). After inclusion of MARS-5 in the model, the direct association remained statistically significant (β = 0.174, SE = 0.079, z = 2.204, *p* = 0.027, 95% bootstrap CI: 0.014 to 0.323). The indirect association through medication adherence was small (β = 0.032, SE = 0.019). Although the corresponding asymptotic maximum-likelihood test did not reach statistical significance (z = 1.665, *p* = 0.096), the bias-corrected bootstrap 95% confidence interval excluded zero (0.005 to 0.082) (Table 7).

Thus, the mediation analysis provided limited, method-dependent evidence of a small indirect association through medication adherence. The direct association between PSPSQ-2 and DLQI remained statistically significant after inclusion of MARS-5, indicating that medication adherence accounted for only a small proportion of their relationship. Given the cross-sectional design, these findings do not establish temporal ordering or causal mediation.

**Figure 2:**
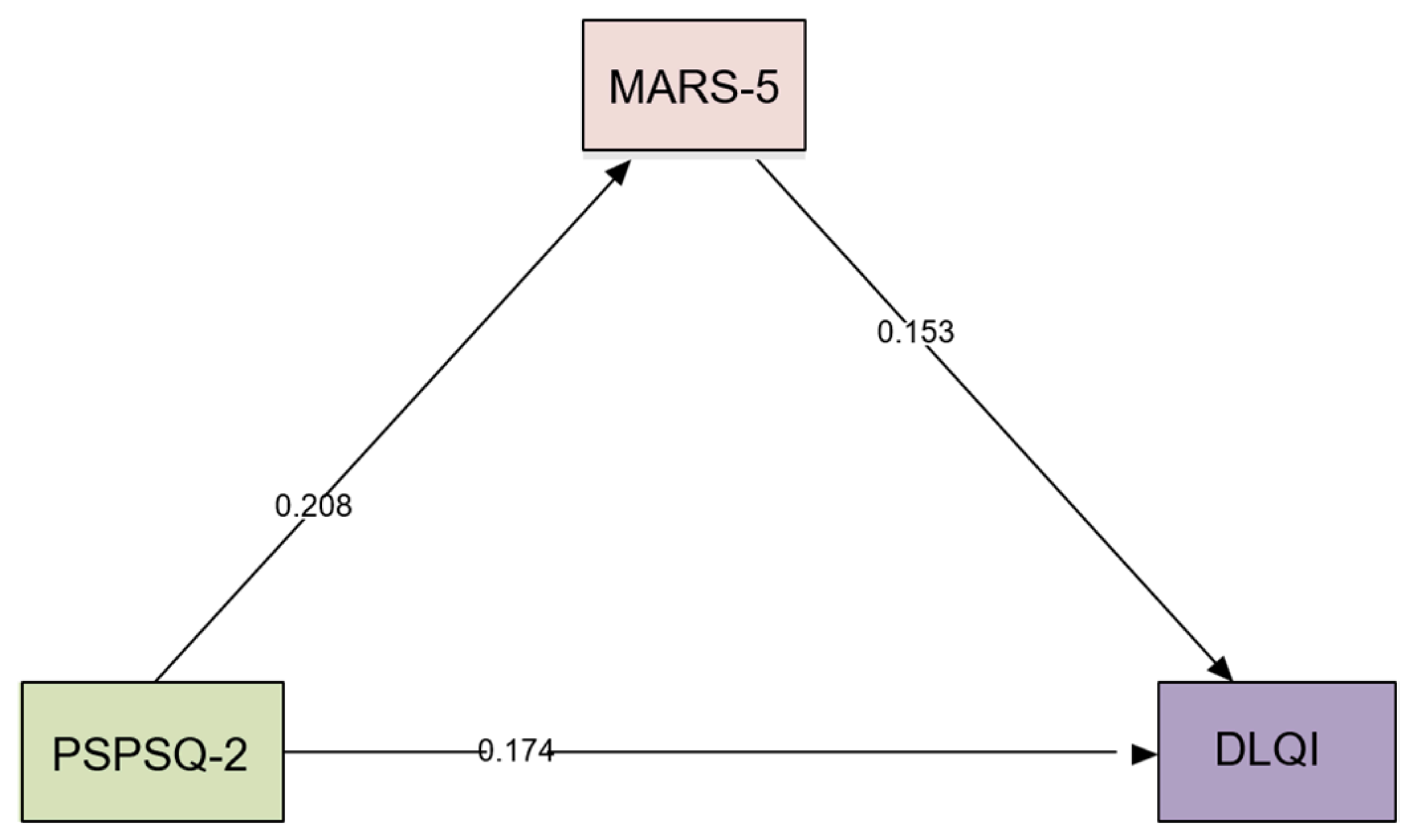
Exploratory mediation model of the relationships among PSPSQ-2, MARS-5, and DLQI. Values represent standardized path coefficients adjusted for age, residence, education, and treatment type. The indirect association through MARS-5 was small; its bias-corrected bootstrap 95% confidence interval excluded zero, whereas the corresponding asymptotic maximum-likelihood test did not reach statistical significance.

The simplified mediation path diagram is shown in Figure 2. PSPSQ-2 was positively associated with MARS-5 (β = 0.208, 95% CI: 0.053 to 0.354, *p* = 0.007), and MARS-5 was positively associated with DLQI (β = 0.153, 95% CI: 0.026 to 0.271, *p* = 0.015). However, the resulting indirect association was small and yielded different conclusions according to bootstrap and asymptotic inference.

### 3.7. Subgroup Analyses

**Figure 3:**
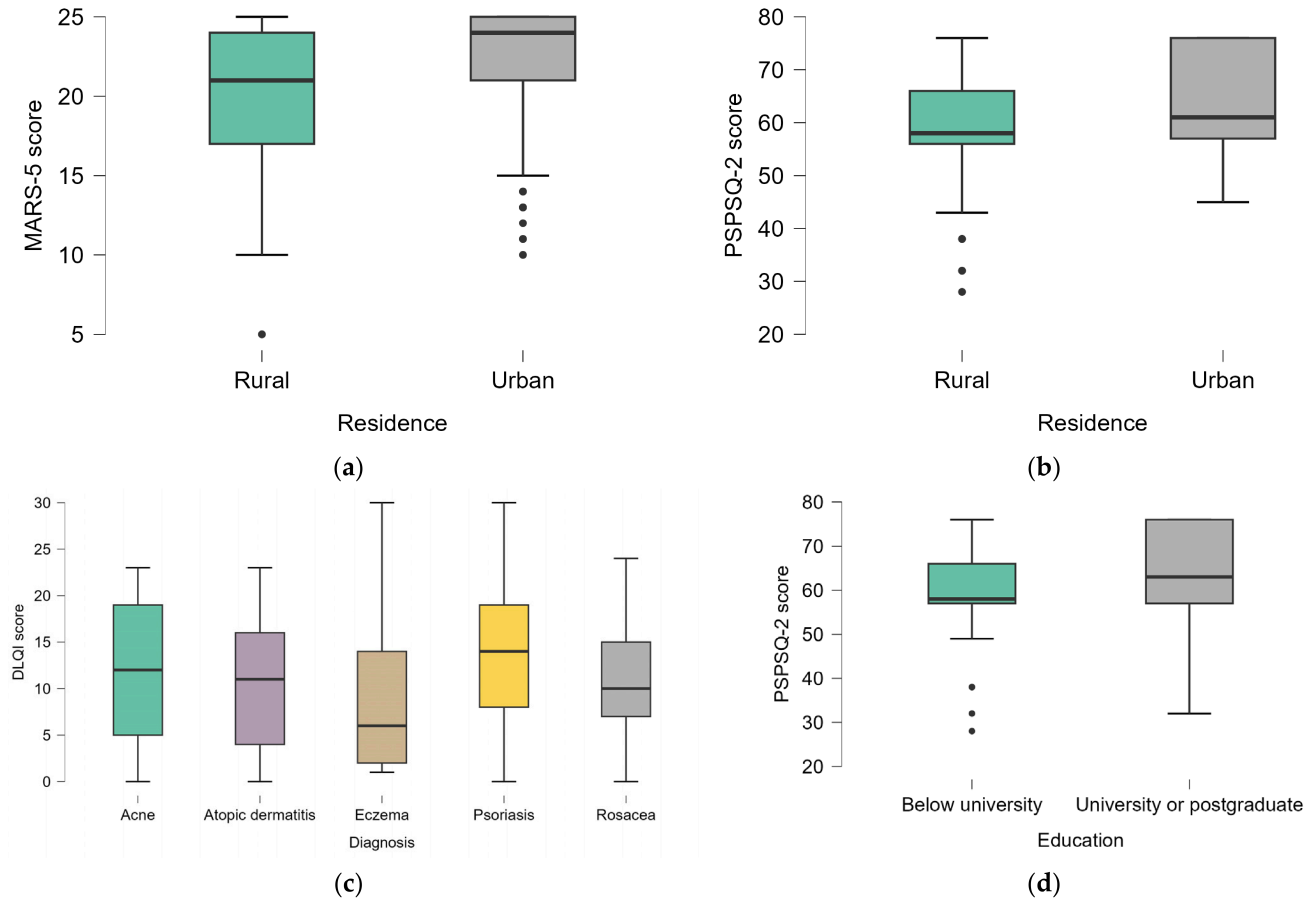
Distribution of MARS-5, PSPSQ-2, and DLQI scores across selected subgroups. (**a**). MARS-5 scores according to residence; (**b**). PSPSQ-2 scores according to residence; (**c**). DLQI scores according to diagnosis; (**d**). PSPSQ-2 scores according to education level. Education was dichotomized as below university versus university/postgraduate. Boxplots show medians, interquartile ranges, and outliers.

Exploratory subgroup analyses were conducted according to residence, diagnosis, and education level. The distributions of MARS-5, PSPSQ-2, and DLQI scores across the analysed subgroups are illustrated in Figure 3.

Medication adherence differed significantly by residence, with higher MARS-5 scores among urban participants than rural participants (Figure 3a; mean 22.28 ± 3.75 vs. 19.91 ± 4.63; Mann–Whitney U test, *p* < 0.001; rank-biserial *r* = −0.353). Satisfaction with pharmacist services was also higher among urban participants (Figure 3b; PSPSQ-2 mean 64.33 ± 8.70 vs. 60.25 ± 10.57; *p* = 0.020; rank-biserial *r* = −0.189), whereas DLQI scores did not differ significantly according to residence (*p* = 0.484).

Significant differences according to diagnosis were observed for DLQI scores (Figure 3c; Kruskal–Wallis *p* = 0.015; rank ε^2^ = 0.058). The highest mean DLQI score was observed among participants with psoriasis (13.55 ± 7.95), whereas the lowest was observed among those with eczema (9.13 ± 8.51). Dunn’s post hoc test demonstrated a significant difference between psoriasis and eczema after correction for multiple comparisons (adjusted *p* = 0.012). Satisfaction with pharmacist services also differed significantly across diagnostic groups in the global Kruskal–Wallis test (*p* = 0.008; rank *ε^2^* = 0.064), although no pairwise comparison remained statistically significant after adjustment for multiple testing. Medication adherence did not differ significantly according to diagnosis (Kruskal–Wallis *p* = 0.054).

When participants were grouped according to educational attainment, satisfaction with pharmacist services was significantly higher among those with university or postgraduate education than among those with lower educational levels (Figure 3d; PSPSQ-2 mean 64.80 ± 9.61 vs. 60.76 ± 9.09; Mann–Whitney U test, *p* = 0.007; rank-biserial *r* = −0.209). Differences in medication adherence and DLQI scores according to education level did not reach statistical significance (*p* = 0.088 and *p* = 0.059, respectively).

## 4. Discussion

Following the publication of the Romanian psychometric validation of the PSPSQ-2, the present cross-sectional study examined the relationships among patient satisfaction with pharmacist services, medication adherence, perceived social support, and dermatology-specific quality of life in Romanian adults with chronic dermatological conditions receiving care in community pharmacies. Greater satisfaction with pharmacist services was associated with better self-reported medication adherence; however, this relationship was attenuated after adjustment for perceived social support. These findings suggest that pharmaceutical care experiences may contribute to treatment-related behaviors, but that their influence operates within a broader psychosocial context. More than half of participants reported very large or extremely large dermatology-related quality-of-life impairment, highlighting the substantial burden associated with chronic skin diseases despite generally high self-reported adherence and satisfaction with pharmacist services.

These mechanisms are consistent with evidence that pharmacist-delivered medication management and favorable pharmacy-related experiences can be associated with better clinical and patient-centred outcomes [8,10]. Patient-centred communication has also been identified as a central component of community pharmacy practice in Romania [27]. Their relevance may be particularly pronounced in dermatology, where treatment regimens may involve prolonged topical therapy, complex application schedules, delayed clinical improvement, and the need for continuing education and reassurance [4].

Perceived social support emerged as the strongest independent correlate of medication adherence in the fully adjusted model. In chronic dermatological conditions, support from family, friends, or the wider social environment may facilitate treatment routines, reinforce motivation, and mitigate the psychological and social consequences of visible skin disease. This interpretation is consistent with evidence across chronic conditions linking social support with medication adherence and self-management behaviors [19,20]. From a pharmaceutical care perspective, the finding suggests that pharmacists may benefit from recognizing psychosocial barriers and available support resources when counselling patients. Where appropriate, pharmaceutical care may incorporate reinforcement of existing support networks or referral to other healthcare resources rather than focusing exclusively on medication instructions. This interpretation is consistent with evidence that community pharmacists already address social determinants of health, although limitations in staff capacity and available resources remain [28].

Substantial dermatology-related quality-of-life impairment was observed despite generally high medication adherence and satisfaction with pharmacist services. This finding indicates that positive healthcare experiences and adherence alone may not fully offset the physical, psychological, and social burden of chronic skin disease. Quality-of-life impairment also varied across the five principal diagnostic groups included in the diagnosis-specific analysis. Participants with psoriasis reported the highest mean DLQI score, and the difference between psoriasis and eczema remained statistically significant after adjustment for multiple comparisons. The greater burden observed in psoriasis may reflect not only clinical manifestations but also stigma, emotional distress, impaired social functioning, and reduced well-being. These observations are consistent with evidence identifying psoriasis as a chronic dermatological condition associated with substantial psychosocial and quality-of-life impairment [23,29,30,31,32]. The findings support the complementary use of patient-reported outcome measures in addition to treatment-related assessments [33,34], while the absence of objective disease-severity indicators prevents determination of whether the observed diagnostic differences reflected clinical severity, psychosocial burden, or both.

Greater engagement with pharmacy services may consequently coexist with poorer health status and higher satisfaction with the support received. More broadly, patient-perceived healthcare quality has been shown to vary according to patients’ health characteristics [35]; however, the proposed explanation remains untested in the present study.

The association between systemic treatment and higher DLQI scores supports this interpretation. Studies involving patients with moderate-to-severe psoriasis or psoriasis treated systemically document substantial residual disease and patient-reported burden [31,36]. In this context, treatment type may act as a marker of disease burden rather than as a cause of reduced quality of life.

The exploratory mediation model suggested a small indirect association between satisfaction with pharmacist services and dermatology-related quality-of-life impairment through medication adherence. The direct association between satisfaction and DLQI remained present after inclusion of MARS-5, indicating that adherence accounted for only a limited proportion of their relationship. Given the cross-sectional design, the model does not establish temporal ordering or causal mediation and should be regarded as hypothesis-generating. Satisfaction primarily reflects patients’ experiences of healthcare delivery, whereas DLQI captures the burden of dermatological disease. Their association may therefore arise through several clinical, behavioral, and contextual pathways that cannot be distinguished in the present analysis. The coexistence of high satisfaction and substantial quality-of-life impairment reinforces the value of integrating patient-reported experience measures and patient-reported outcome measures when evaluating patient-centred care [13,37,38].

Several sociodemographic characteristics provided additional context for the principal findings. Urban residence was associated with higher medication adherence and greater satisfaction with pharmacist services. These differences may reflect greater availability of dermatologists, community pharmacies, treatment-monitoring services, and medication-related information in urban areas. Rural populations may face geographical, organizational, or service-availability barriers that influence treatment continuity and healthcare engagement [39,40]. These findings are consistent with broader evidence that social determinants contribute to differences in health-related quality of life among people with chronic diseases [21].

Higher educational attainment was associated with greater satisfaction with pharmacist services and lower dermatology-related quality-of-life impairment. Patients with higher education may find it easier to understand treatment recommendations, navigate healthcare services, and participate actively in disease self-management, although education should not be considered equivalent to health literacy. Health literacy itself has a modest positive association with adherence to medical treatment [41]. In the fully adjusted adherence model, higher education was associated with slightly lower MARS-5 scores. Because this association was small and was not consistently observed in the bivariate analysis, it should be interpreted cautiously and not regarded as evidence that higher education adversely affects adherence. Overall, the educational findings support the provision of individualized counselling adapted to patients’ comprehension, treatment needs, and health-literacy requirements.

Taken together, the statistically significant bivariate correlations were predominantly weak to moderate, with several sociodemographic associations below |ρ| = 0.20 and perceived social support showing the strongest association with medication adherence. The smaller coefficients indicate modest population-level patterns and have limited explanatory value when considered individually. Moreover, correlation coefficients do not translate directly into clinically meaningful score differences, and no thresholds were prespecified for the clinical importance of these cross-construct associations; therefore, their relevance at the individual patient level cannot be inferred from the present analysis.

By integrating satisfaction with pharmacist services, medication adherence, perceived social support, and dermatology-specific quality of life within a single analytical framework, the present study extends the limited evidence on patient-centred pharmaceutical care in chronic dermatological conditions in Central and Eastern Europe, where clinical pharmacy services remain unevenly developed and efforts to formalize these services are ongoing [22,42]. The findings indicate that favorable experiences with pharmacist services coexist with broader psychosocial and clinical influences on adherence and quality of life. Accordingly, the results support a patient-centred approach in which medication counselling is complemented by attention to treatment burden, psychosocial context, healthcare accessibility, and the need for multidisciplinary referral [28,43]. Because the study was observational and cross-sectional, these implications should be understood as informing future intervention development rather than demonstrating the effectiveness of pharmacist-led interventions.

Key strengths of this study include the simultaneous assessment of satisfaction with pharmacist services, medication adherence, perceived social support, and dermatology-specific quality of life in a real-world community pharmacy setting. The inclusion of patients with various chronic dermatological conditions recruited from multiple pharmacies further enhances the relevance and applicability of the findings.

The findings of the present study should be interpreted in light of several limitations. The cross-sectional design precludes causal inference, and reverse causality cannot be excluded. All primary outcomes were self-reported and may therefore be affected by recall and social desirability bias, particularly medication adherence, which may be overestimated compared with objective measures. The substantial ceiling effects for both MARS-5 and PSPSQ-2 may have reduced score variability and attenuated observed associations. Although the MARS-5 was adapted for dermatological treatments, the adapted version underwent only internal consistency assessment and not full psychometric validation. Perceived social support was assessed using a single self-reported item and therefore may not fully capture the multidimensional nature of social support. In addition, objective measures of disease severity were unavailable. The absence of indicators such as PASI, EASI, SCORAD, or acne severity scores limits interpretation of some findings, particularly those involving treatment type and DLQI. Recruitment from pharmacies located near hospitals and dermatology outpatient clinics may limit generalizability to other pharmacy settings and geographical regions. Eligibility was based on a patient-reported physician diagnosis, corroborated through dermatologist-issued prescriptions and current treatment when available; however, prescription availability was not recorded systematically, and diagnoses were not independently verified against medical records. Finally, the diagnosis-specific subgroup analysis excluded four participants with rare diagnoses because their group sizes were insufficient for reliable comparison, and all subgroup and mediation analyses were exploratory.

Future longitudinal and interventional studies should incorporate objective adherence measures, multidimensional measures of social support, and disease-specific clinical-severity assessments. Such studies are needed to clarify the temporal relationships among pharmaceutical care experiences, treatment behaviors, psychosocial resources, and quality of life and to determine whether pharmacist-led interventions can improve patient-reported and clinical outcomes in chronic dermatological care.

## 5. Conclusions

In this cross-sectional study of Romanian adults with chronic dermatological conditions, greater satisfaction with community pharmacist services was associated with better self-reported medication adherence. However, this association was attenuated after adjustment for perceived social support, highlighting the importance of psychosocial factors alongside pharmaceutical care in supporting long-term treatment engagement.

Substantial dermatology-related quality-of-life impairment was observed despite generally high levels of medication adherence and satisfaction with pharmacist services, emphasizing that positive healthcare experiences do not necessarily reflect lower disease burden. These findings support the complementary value of patient-reported experience and outcome measures in the comprehensive assessment of patients with chronic dermatological conditions.

Overall, the findings underscore the relevance of community pharmacy services within patient-centred dermatological care, particularly in relation to counselling, education, and adherence support. They also indicate that pharmaceutical care should account for the broader psychosocial context, as perceived social support emerged as an important correlate of medication adherence. Further longitudinal and interventional studies are needed to confirm these associations and determine whether pharmacist-led interventions can improve adherence, quality of life, and other patient-centred outcomes in chronic dermatological care.

## Figures and Tables

**Table 1:** Baseline socio-demographic, clinical and access-related characteristics included in the analytical dataset (N = 220).

Characteristic	Category	n (%)
Age group (years)	18–29	55 (25.0)
	30–39	56 (25.5)
	40–49	45 (20.5)
	50–59	37 (16.8)
	≥60	27 (12.3)
Gender	Female	146 (66.4)
	Male	74 (33.6)
Residence	Urban	144 (65.5)
	Rural	76 (34.5)
Education level	Primary	10 (4.5)
	Secondary	92 (41.8)
	University	104 (47.3)
	Postgraduate	14 (6.4)
Occupation	Employed	140 (63.6)
	Student	31 (14.1)
	Retired	31 (14.1)
	Unemployed	11 (5.0)
	Other	7 (3.2)
Marital status	Married	119 (54.1)
	Single	66 (30.0)
	Divorced	28 (12.7)
	Widowed	7 (3.2)
Diagnosis	Eczema	69 (31.4)
	Acne	54 (24.5)
	Psoriasis	51 (23.2)
	Rosacea	29 (13.2)
	Atopic dermatitis	13 (5.9)
	Other	4 (1.8)
Disease duration	<1 year	70 (31.8)
	1–5 years	87 (39.5)
	>5 years	63 (28.6)
Treatment type	Topical only	154 (70.0)
	Systemic (±topical)	59 (26.8)
	Other/phototherapy	7 (3.2)
Healthcare visits	Monthly/every 3 months	19 (8.7)
	Every 6 months	66 (30.0)
	Annually	65 (29.5)
	Rarely	70 (31.8)
Social support	Very good/good	180 (81.8)
	Moderate	26 (11.8)
	Poor/none	14 (6.4)
Access to care	Very easy/easy	197 (89.5)
	Difficult/very difficult	23 (10.5)
Health insurance	Yes	197 (89.5)
	No	23 (10.5)

Other diagnoses comprised seborrheic dermatitis (n = 2) and vitiligo (n = 2).

**Table 2:** Descriptive statistics of primary outcomes at baseline (N = 220).

Measure	Mean ± SD	Median (IQR)	Observed Range	Ceiling (%)
MARS-5	21.50 ± 4.19	23 (19–25)	5–25	35.9
DLQI	11.12 ± 7.86	11 (4–17)	0–30	1.4
PSPSQ-2	62.92 ± 9.57	60 (57–74)	28–76	24.5

**Table 3:** Distribution of participants according to DLQI impact categories at baseline (N = 220).

DLQI Category	Score Range	n (%)
No effect on patient’s life	0–1	29 (13.2)
Small effect	2–5	44 (20.0)
Moderate effect	6–10	33 (15.0)
Very large effect	11–20	87 (39.5)
Extremely large effect	21–30	27 (12.3)

**Table 4:** Statistically significant Spearman correlations between primary outcomes and selected socio-demographic and clinical variables (N = 220).

Variables	*ρ*	*p*
PSPSQ-2—MARS-5	0.232	<0.001
PSPSQ-2—DLQI	0.319	<0.001
MARS-5—DLQI	0.234	<0.001
Social support—MARS-5	0.453	<0.001
Residence—MARS-5	0.288	<0.001
Education—MARS-5	0.137	0.043
Education—PSPSQ-2	0.183	0.006
Residence—PSPSQ-2	0.165	0.014
Age—PSPSQ-2	−0.147	0.030

Only statistically significant correlations involving the primary outcome variables. The complete correlation matrix is shown in Figure 1.

**Table 5:** Multivariable linear regression models with medication adherence, MARS-5, as the dependent variable.

Predictor	Model 1 B	95% *CI*	*β*	*p*	Model 2 B	95% *CI*	*β*	*p*
Residence, urban vs. rural	2.182	0.911 to 3.454	0.248	<0.001	2.057	0.894 to 3.219	0.234	<0.001
PSPSQ-2	0.076	0.017 to 0.135	0.174	0.011	0.054	−0.005 to 0.108	0.122	0.053
DLQI	0.081	0.011 to 0.152	0.153	0.023	0.094	0.030 to 0.159	0.177	0.004
Gender	0.117	−1.018 to 1.253	—	0.839	0.059	−0.979 to 1.096	—	0.911
Age	0.016	−0.025 to 0.056	0.051	0.446	0.019	−0.018 to 0.055	0.062	0.313
Education	−0.121	−0.730 to 0.487	−0.029	0.695	−0.601	−1.176 to −0.027	−0.143	0.040
Social support	—	—	—	—	1.787	1.251 to 2.323	0.411	<0.001

Model 1 adjusted for residence, satisfaction with pharmacist services, DLQI, gender, age and education. Model 2 additionally included perceived social support. Higher DLQI scores indicate greater dermatology-related quality-of-life impairment. Education was dichotomized as university/postgraduate versus below university. Perceived social support was entered as the original five-level ordinal score, with higher values indicating greater perceived support. PSPSQ-2, Patient Satisfaction with Pharmacist Services Questionnaire 2.0; MARS-5, Medication Adherence Report Scale; DLQI, Dermatology Life Quality Index.

**Table 6:** Multivariable linear regression model with dermatology-related quality-of-life impairment, DLQI, as the dependent variable (N = 220).

Predictor	*B*	95% *CI*	*β*	*p*
Treatment type	3.110	0.823 to 5.398	0.176	0.008
PSPSQ-2	0.175	0.066 to 0.284	0.213	0.002
Gender	−0.285	−2.431 to 1.861	−0.017	0.794
Age	−0.050	−0.124 to 0.025	−0.087	0.188
Education	−1.508	−2.544 to −0.473	−0.192	0.005

Higher DLQI scores indicate greater dermatology-related quality-of-life impairment. Treatment type was dichotomized as systemic treatment, with or without topical therapy, versus non-systemic treatment. Education was dichotomized as university/postgraduate versus below university. PSPSQ-2, Patient Satisfaction with Pharmacist Services Questionnaire 2.0; DLQI, Dermatology Life Quality Index.

**Table 7:** Exploratory mediation analysis examining medication adherence as a potential mediator of the association between satisfaction with pharmacist services and dermatology-related quality-of-life impairment.

Effect	Estimate	*SE*	*z*	*p*	95% Bootstrap *CI*
Total effect: PSPSQ-2 → DLQI	0.206	0.077	2.676	0.007	0.050 to 0.351
Direct effect: PSPSQ-2 → DLQI	0.174	0.079	2.204	0.027	0.014 to 0.323
Indirect effect: PSPSQ-2 → MARS-5 → DLQI	0.032	0.019	1.665	0.096	0.005 to 0.082

Estimates are standardized coefficients. Confidence intervals were obtained using bias-corrected percentile bootstrap resampling based on 20,000 resamples. The reported *p*-values are based on asymptotic maximum-likelihood z-tests. The model was adjusted for age, residence, education, and treatment type.

## Data Availability

The data presented in this study are available on request from the corresponding author upon reasonable request, subject to ethical considerations and approval of the request. The data are not publicly available due to ethical and research-related questions.

## Abbreviations

The following abbreviations are used in this manuscript:

CI	Confidence interval
DLQI	Dermatology Life Quality Index
EASI	Eczema Area and Severity Index
IQR	Interquartile range
MARS-5	Medication Adherence Report Scale-5
PASI	Psoriasis Area and Severity Index
PREMs	Patient-reported experience measures
PROMs	Patient-reported outcome measures
PSPSQ-2	Patient Satisfaction with Pharmacist Services Questionnaire 2.0
SCORAD	Scoring Atopic Dermatitis
SD	Standard deviation
VIF	Variance inflation factor
